# Abdominal Inflammatory Myofibroblastic Tumour Presenting as a Pancreatic Mass: A Case Report

**DOI:** 10.7759/cureus.41213

**Published:** 2023-06-30

**Authors:** Afafe Taiymi, Nasiri Meryem, Mohammed Bouziane, Abdelkrim Zazour, Ghizlane Kharrasse, Wafaa Khannoussi, Zahi Ismaili

**Affiliations:** 1 Gastroenterology and Hepatology, Digestive Disease Research Laboratory, Mohammed First University, Oujda, MAR; 2 Gastroenterology and Hepatology, Mohammed VI University Hospital, Oujda, MAR; 3 General Surgery, Mohammed VI University Hospital, Oujda, MAR; 4 Gastroenterology and Hepatology, Digestive Disease Research Laboratory, Oujda, MAR

**Keywords:** case report, surgery, myofibroblastic, pseudotumor, inflammatory

## Abstract

An abdominal inflammatory myofibroblastic tumor (AIMT), is a rare benign tumor composed of inflammatory and other mesenchymal cells. It can affect the entire body, predominantly in children and young adults. The diagnosis is challenging considering the wide clinical presentation and can often be mistaken for malignant tumors.

We report a rare case of a 46-year-old female patient, who presented with intermittent abdominal pain weight loss, and an abdominal palpable mass. Abdominal ultrasound found a well-defined 18 cm, rounded mass, with solid and cystic components. Abdominal CT demonstrated a well-defined, hypodense, retro gastric mass of 20 cm, with thickened wall and heterogenous enhancement. The mass had contact with the pancreatic tail, transverse colon, spleen, left kidney pedicles, abdominal aorta, superior mesenteric vein, and mesaraic trunk with no invasion signs. The mass was initially thought to be pancreatic cancer, but given the large size, other diagnoses like sarcoma, lymphoma, or abdominal hydatid cyst were suggested. Endoscopic ultrasound found a rounded retro gastric mass of 18/12 cm, with a thickened wall and well-limited calcifications. The content was both cystic and solid with mobile vegetations, with no visible Doppler flow. The mass had contact with the body and tail of the pancreas, spleen hilum, the upper pole of the spleen, and the hepatic pedicle behind, with no invasion sign. After a multidisciplinary team meeting, a decision was taken to perform surgical resection with mass resection, distal splenopancreatectomy, and transverse and sigmoid colectomy. Pathological and immunostaining results were consistent with inflammatory pseudotumor. The postoperative recovery was uncomplicated. The patient remains asymptomatic with no obvious signs of metastasis or recurrence.

AIMT represents a reel diagnostic challenge. Clinical symptoms are unspecific. Radiological and endoscopic features can often be mistaken for malignant tumors. Surgical management remains to be the best therapeutic option. We report a rare case of AIMT treated by surgery with complete resection. We suggested a long-term follow-up given the local recurrence risk.

## Introduction

An inflammatory myofibroblastic tumor (IMT), also known as an inflammatory pseudotumor or myofibroblastic pseudotumor, is a rare benign tumor composed of various inflammatory and mesenchymal cells [1]. It can affect the entire body and occurs predominantly in children and young adults [2]. The diagnosis is challenging given the wide clinical presentation and can often be mistaken for malignant tumors. We report a clinical case of abdominal IMT (AIMT) presenting as a pancreatic mass with a review of the relevant literature.

## Case presentation

**Figure 1:**
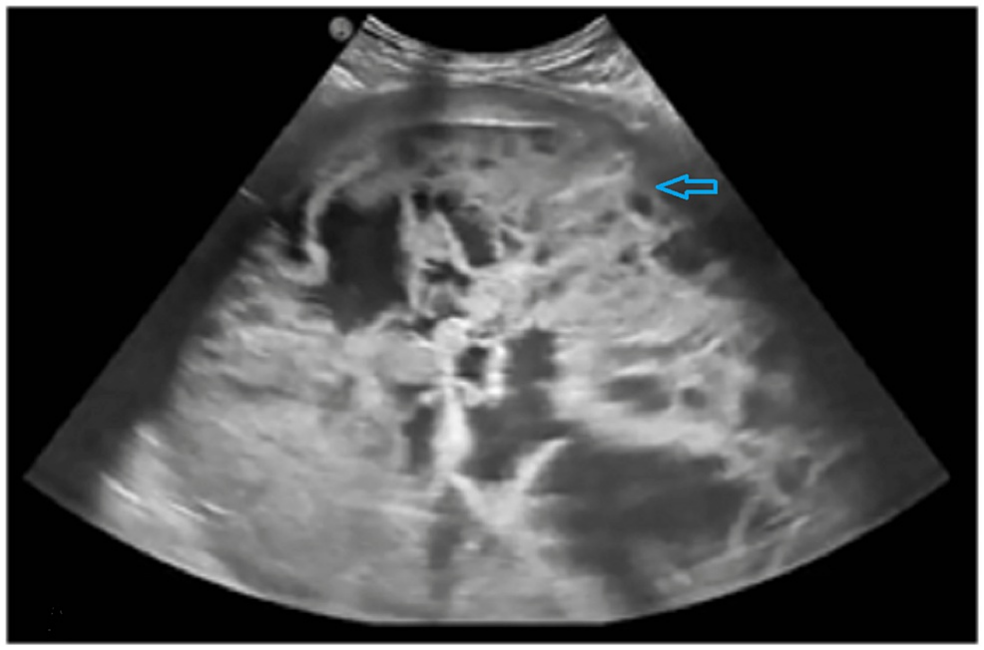
Abdominal ultrasound image Ultrasound showed a well-defined, heterogeneous, rounded abdominal mass, with solid and cystic components.

**Figure 2:**
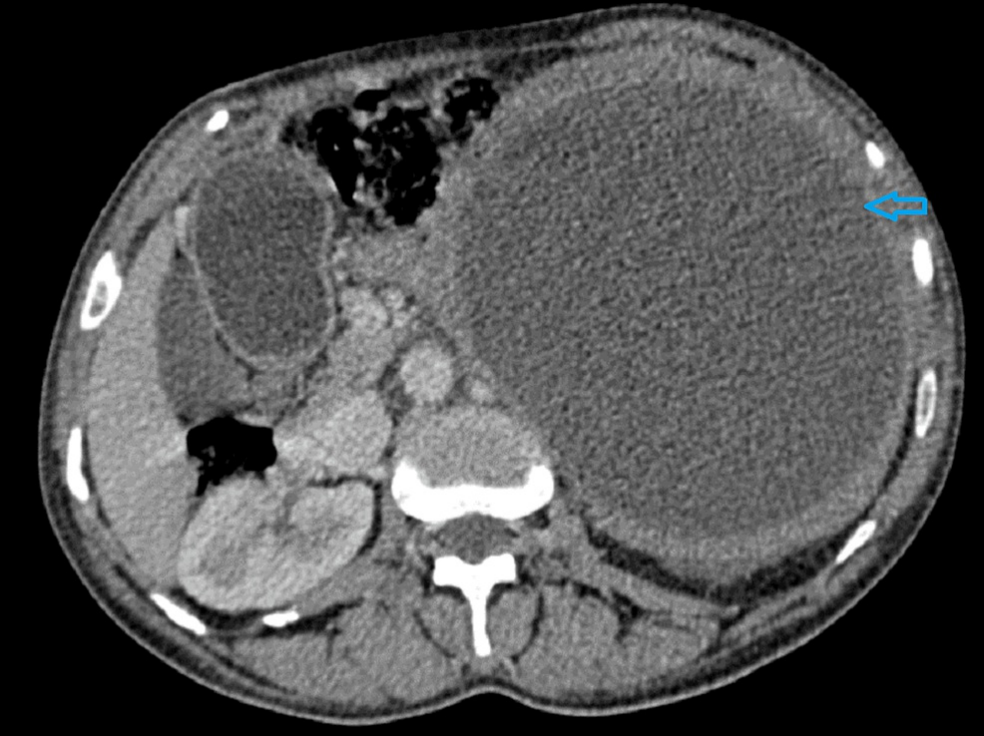
Abdominal computed tomography image The image is showing a well-defined, hypodense, retrogastric 20-cm mass with thickened wall and heterogenous enhancement.

A 46-year-old woman without any past medical history was referred to our gastroenterology unit in February 2022 to evaluate an intra-abdominal mass detected by ultrasound in a nearby hospital. The patient had intermittent abdominal pain for three months. She reported a weight loss of 2 Kg over the past two months. Physical examination revealed a palpable mass of 18 cm, extending from the left hypochondrium to the left flank. Laboratory results, including complete blood count, C-reactive protein, and tumor markers, were all within reference ranges. Abdominal ultrasound found a well-defined, heterogeneous, rounded abdominal mass with a solid and cystic component measuring 18 cm (Figure 1). Abdominal computed tomography (CT) demonstrated a well-defined, hypodense retrogastric mass of 20 cm with thickened wall and heterogeneous enhancement (Figure 2). The mass was in contact with the pancreatic tail, transverse colon, spleen, left kidney pedicles, abdominal aorta, superior mesenteric vein, and mesaraic trunk without invasion signs. The mass origin was difficult to specify, given its size. Several differential diagnoses were suggested, including pancreatic adenocarcinoma, sarcoma, lymphoma, spleen epithelial cyst, and abdominal hydatid cyst.

**Figure 3:**
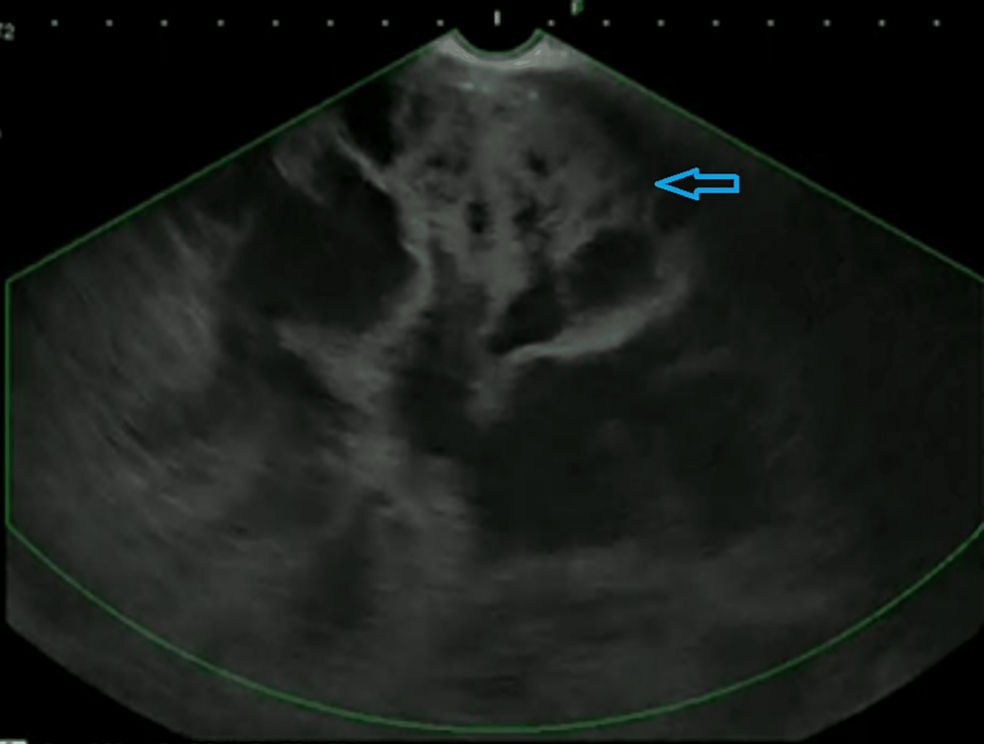
Endoscopic ultrasound image Endoscopic ultrasound showed a rounded retrogastric mass with thickened wall. The content was both cystic and solid with mobile vegetation.

Endoscopic ultrasound (EUS) revealed a round retrogastric mass of 18 cm by 12 cm, with flaky and well-limited calcifications; the content was both cystic and solid with mobile vegetation. It had no visible Doppler flow, and the mass had contact with the body and tail of the pancreas, the spleen hilum, the upper pole of the spleen, and the hepatic pedicle behind without invasion sign. The mass was in contact with the gastric wall with no invasion sign (Figure 3).

**Figure 4:**
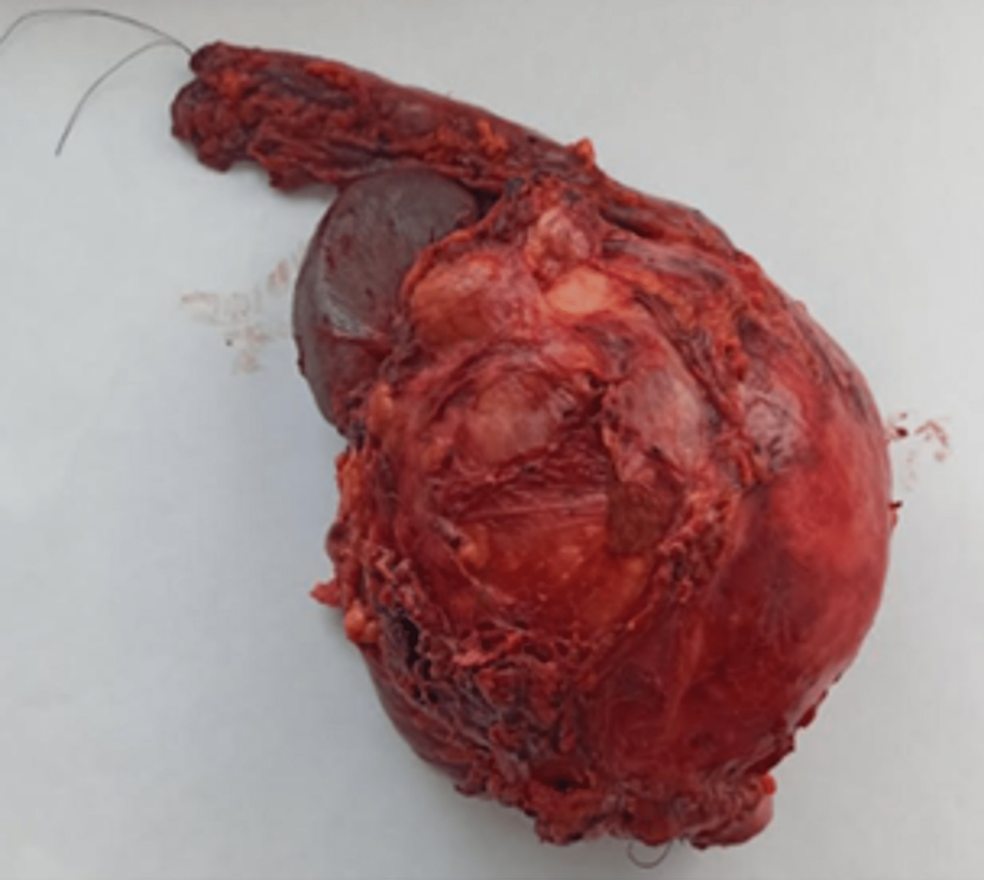
A large 20-cm mass completely resected by laparotomy

After a multidisciplinary team meeting, the decision was made to perform a laparotomy for diagnosis and treatment. Exploration revealed a large cystic mass of 20 cm in close contact with the tail pancreas, spleen, left kidney, transverse, and sigmoid colon, with multiple peritoneal adhesions. No liver lesions or peritoneal carcinomatosis were detected. It was decided to perform the mass resection, with distal splenopancreatectomy and transverse and sigmoid colectomy (Figure 4).

Macroscopically, the mass had a cystic aspect with dark fluid content, thickened fibrous wall, and vegetation. Histological study revealed a well-limited myofibroblastic tumor proliferation, with bundles of regular fibroblasts and clusters of dense collagen. Congestive vessels and inflammatory infiltrate of lymphocytes, plasma cells, and eosinophils were detected. Tumor proliferation adheres intimately to the pancreas, spleen, and transverse and sigmoid colon with no invasion signs. The immunohistochemical study stained negatively for PS100, STAT6, ALK, desmin, CD117, and CD34. A diagnosis of an intraabdominal pseudo-inflammatory tumor was determined, given the histopathological results. The patient’s postoperative recovery was uncomplicated. She was discharged from the hospital on day seven. The patient is asymptomatic with no obvious signs of metastasis or recurrence over the 15-month follow-up.

## Discussion

IMT is rare and benign and often occurs in young patients [1,2]. AIMT is extremely rare and includes the retroperitoneum, pancreas, liver, mesentery, kidney, esophagus, stomach, small intestine, and colon [3-5]. While the etiology of AIMT is unknown, several causes are suspected, including infection with the Epstein-Barr virus, cytomegalovirus, mycobacteria, trauma, irradiation, or surgery [6,7]. Our patient had no past medical history and none of these etiologies. Although AIMTs are benign neoplasms, a rare incidence of malignant transformation has been reported [7]. World Health Organization Classification of Soft Tissue and Bone currently classifies IMT as intermediate neoplasms [8,9]. The clinical symptoms vary according to the involved organ. Although digestive symptoms in AIMT are nonspecific, patients can experience abdominal discomfort, dyspepsia, weight loss, and palpable mass (as was seen in our case). Laboratory findings are nonspecific, and iron deficiency anemia can be detected [10]. The similarities with malignant tumors represent a diagnostic challenge. Additionally, the radiological characteristics of AIMT are not specific. After CT contrast enhancement, AIMT can appear as a large heterogeneous mass with necrotic hypodense areas, well-defined margins, and well-vascularized solid tumors [11,12]. Calcifications and fatty components have been described in nonenhancement CTs [12]. In our case, an ultrasound found a well-defined heterogeneous, large abdominal mass, and abdominal CT demonstrated a well-defined, hypodense retrogastric mass of 20 cm, with heterogeneous enhancement.

EUS is performed for better diagnostic accuracy if radiological results were inconclusive for subsequent treatment. The role of EUS in diagnosing pancreatic masses, non-pancreatic, and retroperitoneal lesions is well established [13]. Erikson et al. found that EUS can provide superior results to CT about the precise origin of retroperitoneal masses and/or its involvement of adjacent organs in 38% of cases [14].

However, the diagnosis of AIMT in EUS remains challenging, and fine needle aspiration biopsy is ineffective [15]. A surgical approach is usually indicated for a definitive histological diagnosis [15,16]. AIMT is characterized histologically by myofibroblastic spindle cells and inflammatory cells, such as lymphocytes, histiocytes, and eosinophils in the collagenated stroma [17]. Molecular aberrations of anaplastic lymphoma kinase (ALK) receptors on chromosome 2p23 were found in 50% to 70% of AIMT cases [18]. Therefore, AIMT is a neoplasm with specific histological characteristics of inflammatory sclerosing and fibrosing processes [18]. However, no relationship has been established between ALK expression and prognosis [9]. In an immunohistochemical study, AIMT is positive for actin, desmin, vimentin, CD68, and CD34 [3].

Surgery with complete resection is recommended as the first therapeutic option of AIMT. Chemotherapy and radiotherapy have limited roles as adjunctive treatments. High-dose steroids or imatinib may be indicated in inoperable tumors [9]. AIMT is regarded as an intermediate neoplasm with a favorable prognosis [3]. However, local recurrence after surgery occurs in 15% to 37% of cases, which requires long-term follow-up [19,20]. In our series, the patient had surgery with complete tumor resection after a multidisciplinary team discussion. No postoperative treatment was administered, and the prognosis was favorable.

## Conclusions

This case highlights that AIMTs are a diagnostic challenge because they usually mimic malignant tumors. Surgical management remains the best therapeutic option. The prognosis is often favorable but requires long-term monitoring, given the risk of recurrence. The case presented was treated by surgery with complete resection and a long-term follow-up was suggested considering the local recurrence risk.
